# Some Diagnostic Remarks

**Published:** 1888-11

**Authors:** J. M. Miller


### SOME DIAGNOSTIC REMARKS.

J. M. MILLER, M. D.

*Silicea* has a symptom: Palpitation while sitting quietly, so that the hand in which he is holding something trembles. Hahnemann giving *Silicea* according to other symptoms, cured an unpainful beating, throbbing, and hammering in the breastbone with it. My old friend Brauns, in Thuringia, was induced to give *Silicea* to a boy of twelve years, suffering by spells with a most violent hammering palpitation, "endangering his life." *Aconite* only lessened it a little, and *Sepia* seemed to overcome the remaining hard shocks of the heart; still the attacks returned, and *Aurum*, given by the parents, did nothing. Finding that the boy was always attacked after quick or violent motion, playing ball, etc., Brauns ordered *Sil.*$^{30}$. Even six months after, the boy had not had another attack. Later, Brauns found out that the boy had been subject to panaritia, and at times they had been checked by washing with water from the tub used by a blacksmith to cool the hot iron. Thus the *Silicea* had been indicated before, in this case. According to Goullon, the pains of *Silicea* are increased by exercise, but with palpitation we did not know it until Brauns published his case. He had several cases similar to the one quoted, and other physicians have corroborated the same symptom. *Silicea* is not mentioned in any of the cases collected by Ruckert and Oehme, in the chapter on Diseases of the Heart. In all cases cured by *Silicea* alone, there were no signs of an organic disease, nevertheless it could have come to it. In some cases with decided organic disease, *Silicea* was of great service.

*Phosphorus.* A boy of nine years got in the morning, without any known cause, and without any objective signs of congestion, a most violent hammering in the chest, which was aggravated by every motion. In the afternoon, at three o'clock, while walking across the yard, it increased so that it benumbed him, and he fell and wounded his face on the right cheek-bone. Brauns allowed him to smell of *Phosphorus*$^{30}$, and the hammering ceased. The wound was washed with water, containing *Arnica*$^{30}$. It was covered by a crust next day. Four months after he had not had a return of the hammering.

Palpitation brought on and indicated by the slightest motion, indicates *Spigelia*—that is, if the other symptoms correspond. We know this from cured cases, not from the symptoms of the provers. Moving the arms causes palpitation of the heart, may indicate *Digitalis.*

*Ferrum* has anxiety in the chest, and heat rising from the pit of stomach upwards, after bodily exercise, and is often indicated in heart disease.

Bœnninghausen's experience is lost, because he has never separated the conditions of increase and decrease of symptoms in the chest and the heart. He gives in his Old Repertories of 1833 and 1835, a long list of remedies, having increase by motion, of the internal or external symptoms of the chest or the heart, or the mammæ, etc., of which we will mention here, only the most important.

The foremost are *Calcarea*, *Phosphorus*, and *Bryonia.* The next *Graph.*, *Senega*, *Sepia*, *Zincum*, and *Acon.*, *Arn.*, *Ledum*, *Nux vom.*, *Ran. bulb.* Less important, *Bell.*, *Merc.*, *Camph.*, *Caps.*, *China*, *Colch.*, *Puls.*, *Staph.*; in fact, all the Ranunculaceæ have it. *On moving arms*, besides *Digitalis: Ledum*, *Nux mosch.*, *Nux vom.*, *Plumbum*, *Puls.*, *Ran. bulb.*, and *Viola tricolor.*

